# CD164 promotes lung tumor-initiating cells with stem cell activity and determines tumor growth and drug resistance via Akt/mTOR signaling

**DOI:** 10.18632/oncotarget.11132

**Published:** 2016-08-09

**Authors:** Wei-Liang Chen, Ai-Fang Huang, Shih-Ming Huang, Ching-Liang Ho, Yung-Lung Chang, James Yi-Hsin Chan

**Affiliations:** ^1^ Graduate Institute of Medical Sciences, National Defense Medical Center, Taipei 114, Taiwan, Republic of China; ^2^ Division of Family Medicine, Department of Family and Community Medicine, Tri-Service General Hospital, and School of Medicine, National Defense Medical Center, Taipei 114, Taiwan, Republic of China; ^3^ Division of Geriatric Medicine, Department of Family and Community Medicine, Tri-Service General Hospital, and School of Medicine, National Defense Medical Center, Taipei 114, Taiwan, Republic of China; ^4^ Department of Biochemistry, National Defense Medical Center, Taipei 114, Taiwan, Republic of China; ^5^ Division of Hematology, Department of Medicine, Tri-Service General Hospital, National Defense Medical Center, Taipei 114, Taiwan, Republic of China; ^6^ Department of Microbiology and Immunology, National Defense Medical Center, Taipei 114, Taiwan, Republic of China; ^7^ Department of Medical Research, Tri-Service General Hospital, National Defense Medical Center, Taipei 114, Taiwan, Republic of China

**Keywords:** CD164, lung cancer, tumorigenesis, cancer stem cell, drug resistance

## Abstract

CD164 is a cell adhesion molecule that increases hematopoietic stem cell proliferation, adhesion, and migration via C-X-C chemokine receptor type 4 (CXCR4) signaling. Emerging evidence indicates that elevated CD164 expression is associated with aggressive metastasis, advanced stages, and shorter overall survival in lung cancer. However, no data are available regarding the clinical significance of CD164 expression in lung cancer. This study explores whether CD164 promotes tumor-initiation and drug resistance through the stem cell property. Using tissue microarrays, we determine that CD164 expression is correlated with clinicopathological characteristics in human lung cancer. The CD164 overexpression in normal lung epithelial cells (BEAS2B cells) leads to malignant transformation *in vitro*, tumorigenicity in xenografted mice, stem cell-like property, and drug resistance through ATP-binding cassette transporters. The CD164 overexpression increases CXCR4 expression and activates Akt/mTOR signaling. Rapamycin, an mTOR inhibitor, hinders cell proliferation along with sphere formation *in vitro* and impedes tumor growth *in vivo*. In conclusion, we have provided evidence that CD164 promotes the growth of lung tumor-initiating cells with stem cell properties and induces tumor growth and drug resistance through Akt/mTOR signaling. Therefore, identification of CD164 as a cancer stem cell therapeutic marker may develop an effective therapy in patients with chemoresistant lung cancer.

## INTRODUCTION

Lung cancer has rapidly emerged as the leading cause of death among men and women worldwide over the past several decades and is mainly categorized into small cell lung cancer (SCLC) and non-small cell lung cancer (NSCLC) [1]. In 2004, an estimated 173,700 Americans receive a diagnosis of lung cancer, and 164,440 of them will die of the disease [2]. In spite of advances in multimodal therapy, lung cancer remains a life-threatening disease with a poor actual 5-year survival rate in the range of 10-15% during late-stage disease [3]. Unsatisfactory prognosis was attributed to the presence of more advanced stages at initial diagnosis and high recurrences with resistance to chemotherapy.

Cancer stem cells (CSCs), or tumor-initiating cells, represent a small distinct population of cancer cells and share common properties with normal stem cells. CSCs have multiple unique features that cause them to be vital for tumors growth, such as self-renewal capacity, specific surface biomarker phenotypes, spheroid morphology, and radio-chemotherapy resistance [4]. Current radio- and chemotherapies destroy the bulk of cancer cells, but fail to eliminate critical CSCs that are protected by specific resistance mechanisms [5]. Accumulating evidences indicate therapeutic resistance of lung cancer by the exclusive ability to perpetuate the growth of tumor-initiating cells, which is responsible for tumor progression, recurrence, and metastasis [6, 7]. Over the last few decades, increasing numbers of studies have shed more light on specific surface biomarkers of CSCs in lung cancer, including CD44 [8], CD133 [9], aldehyde dehydrogenase 1 (ALDH1) [10], C-X-C chemokine receptor type 4 (CXCR4) [11], and ABCG2 [12]. The expression of several important CSCs biomarkers also portends a dismal prognostic survival and treatment resistance [13, 14]. Notably, Zhang and his colleagues determine that no significant differences in the xenograft-initiating capacities of CD133^+^ or CD44^+^ tumor cells in comparison to CD133^−^ or CD44^−^ tumor cells in a mouse mode [15]. This implies that CSCs have been shown to be very heterogeneous and possess independently specific markers for each subpopulation. Exploring promising biomarker signatures of CSCs and understanding of complexity of signaling networks may facilitate the development of more effective targeted therapies for lung cancer.

CD164 is a novel 80 to 100-kDa type 1 transmembrane sialomucin and is highly expressed on primitive CD34^+^ hemopoietic progenitor cells [16, 17]. CD164 acts as a component of CXCR4 complexes and increases human umbilical cord blood CD133^+^ cell migration [18]. CD164 appears to be involved in regulating proliferation, adhesion, and migration of hematopoietic progenitor cells, and in maintenance of the undifferentiated state of progenitor cells [18, 19]. CD164 promotes colon cancer cell proliferation and metastasis *in vitro* and *in vivo* possibly through the CXCR4 pathway [20]. The above-mentioned results provide that CD164 may be a cancer promoting gene associated with tumorigenesis.

One meta-analysis of the relationship between CXCR4 expression and lung cancer indicates that elevated CXCR4 expression is correlated with aggressive metastasis, advanced TNM stages, and shorter overall survival in NSCLC patients, suggesting a poor prognostic outcome of this disease [21]. In addition, our previous study demonstrates that CD164 activates CXCR4 and its downstream pathway [22]. We investigate whether the functional roles of CD164 promote lung tumor-initiation and drug resistance through the Akt/mTOR axis, as the clinical significance of CD164 expression in lung cancer has not been reported to date.

## RESULTS

### CD164 expression in human lung cancer and its correlation with clinicopathological characteristics

**Figure 1:**
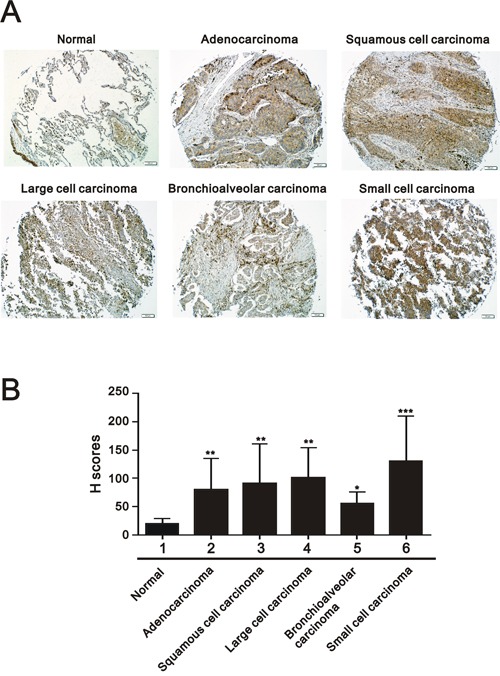
CD164 expression in different clinicopathological parameters of lung cancer **A**. Representative immunohistochemical CD164 staining of lung cancer. **B**. Quantitative analysis of immunohistochemical staining using H-score. H scores of these groups were analyzed using ANOVA. *P < 0.05, **P < 0.01 and ***P < 0.001 versus the normal lung tissues.

To determine the difference in CD164 expression between normal lung tissue and lung cancer tissue, two sets of tissue microarrays including normal lung tissues and cancer tissues of different histological grades and clinical stages were performed for immunohistochemical staining. As shown in Figure 1A, CD164 was mainly expressed in the cytoplasm and membrane of normal lung tissues and lung cancer tissues. Among lung cancer tissues, the tumors demonstrated heterogeneous staining patterns. Different lung cancer cells, including adenocarcinoma, squamous cell carcinoma, large cell carcinoma, and small cell lung cancer, exhibited significantly higher mean CD164 H-scores than normal lung cells (Figure 1B). CD164 immunohistochemistry revealed the existence of significantly positive associations between CD164 expression and tumor size (p=0.001), lymph node involvement (p=0.001), and tumor cell grading (p=0.043) (Table 1). CD164 expression was not significantly associated with other clinical characteristics, such as age, sex, and the presence of metastasis.

**Table 1:** Correlation between the clinical characteristics and the immunohistochemical expressions of CD164 in patients with lung cancer

Clinical characteristics		Number of patients (%)	CD164 expression	
			H-score (Mean ± SD)	p-value
**Age**	<60	175 (48.6 %)	86.1 ± 62.3	
	≧60	185(51.4 %)	95.1 ± 67.0	0.188
**Sex**	Male	297(82.5 %)	93.0 ± 65.7	
	Female	63(17.5 %)	80.1 ± 60.3	0.153
**Tumor cell type**	Normal	20 (5.2%)	20.3 ± 15.6	
	Others	23(6.4 %)	81.3 ± 45.6	0.001
	Adenocarcinoma	138(38.3 %)	80.1 ± 55.1	
	Squamous cell carcinoma	150(41.7%)	91.3 ± 69.5	
	Large cell carcinoma	12(3.3 %)	102.2 ± 51.7	
	Small cell lung cancer	37(10.3 %)	131.1 ± 78.5	
**TNM staging**				
**T**	1	34(9.5 %)	61.7 ± 57.5	
	2	261(72.7 %)	82.8 ± 61.6	0.001
	3 and 4	64(17.8 %)	139.5 ± 58.2	
**N**	0	130(42.6 %)	60.7 ± 50.9	
	1 and 2 and 3	175(57.4%)	117.1 ± 68.1	0.001
**M**	0	353(98.3 %)	90.2 ± 64.5	
	1	6(1.7 %)	136.3 ± 76.4	0.084
**Grade**	1	55(19.7 %)	74.1 ± 47.4	
	2 and 3	224(80.3 %)	90.0 ± 66.1	0.043

Others (Tumor cell type) included bronchioalveolar carcinoma (n=7), adenosquamous carcinoma (n=5), atypical carcinoid (n=4), mucinous carcinoma (n=4), papillary adenocarcinoma (n=2), and sarcomatoid carcinoma (n=1).

### CD164 overexpression alters cell morphology and induces malignant transformation in normal lung epithelial cell, BEAS2B cell

**Figure 2:**
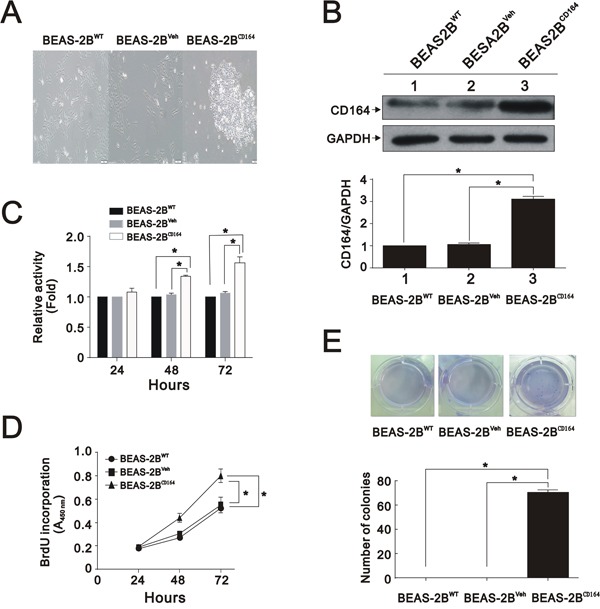
*In vitro* characterization of BEAS2B^CD164^ cells **A**. Cellular morphology of BEAS2B^CD164^ cells compared with BEAS2B^WT^ and BEAS2B^Veh^ cells by microscopy. **B**. Immunoblotting analysis showed CD164 expressions in BEAS2B^CD164^ cells, BEAS2B^WT^ cells, and BEAS2B^Veh^ cells. The results were the means ± SEMs of three independent experiments. *P < 0.05 indicated statistical significance as compared with BEAS2B^Veh^ cells. **C**. Cell viability of BEAS2B^CD164^ cells, BEAS2B^WT^ cells, and BEAS2B^Veh^ cells were analyzed by the MTT assay. The results were the means ± SEMs of three independent experiments. **D**. Proliferation of BEAS2B^CD164^ cells, BEAS2B^WT^ cells, and BEAS2B^Veh^ cells were evaluated by the BrdU proliferative assay. The results were the means ± SEMs of three independent experiments. **E**. Effect of CD164 overexpression on anchorage independent growth. Quantitative analysis of soft agar colony formation assay was performed. The results were the means ± SEMs of three independent experiments. *P < 0.05 indicated statistical significance as compared with BEAS2B^Veh^ cells and BEAS2B^WT^ cells.

The correlation between CD164 expression and tumor stage prompted us to investigate whether CD164 increased malignant transformation in normal lung epithelial cell, BEAS2B cells. We transfected BEAS2B cells using a human CD164 cDNA construct, resulting in the overexpression of CD164. After examining the cellular morphology of CD164-overexpression BEAS2B cells (BEAS2B^CD164^) by phase-contrast microscopy, spindle shaped to small polygonal cell and cell aggregation were observed (Figure 2A). Compared with parent BEAS-2B cell (BEAS2B^WT^) and BEAS2B-vehicle cell (BEAS2B^Veh^) (BEAS2B transfected with empty vector), BEAS2B^CD164^ cells exhibited a 3-fold increase of CD164 expression, as determined by western blotting analysis (Figure 2B). Regarding cell proliferation rates examined by MTT assay, BEAS2B^CD164^ cells showed significantly higher proliferation rates than BEAS2B^WT^ and BEAS2B^Veh^ cells (Figure 2C). Consistent with these results, DNA synthesis was significantly increased in BEAS2B^CD164^ cells in comparison to BEAS2B^WT^ and BEAS2B^Veh^ cells using the BrdU incorporation assay (Figure 2D). To determine the effects of CD164 expression on anchorage-independent growth, cell colony formation was recorded after 4 weeks of incubation in soft agar. We noted more BEAS2B^CD164^ cell colonies than BEAS2B^WT^ and BEAS2B^Veh^ cell colonies (Figure 2E). These findings indicated that CD164 overexpression promoted BEAS2B cell proliferation and anchorage-independent growth.

### CD164 overexpression promotes tumorigenicity in xenografted mice

**Figure 3:**
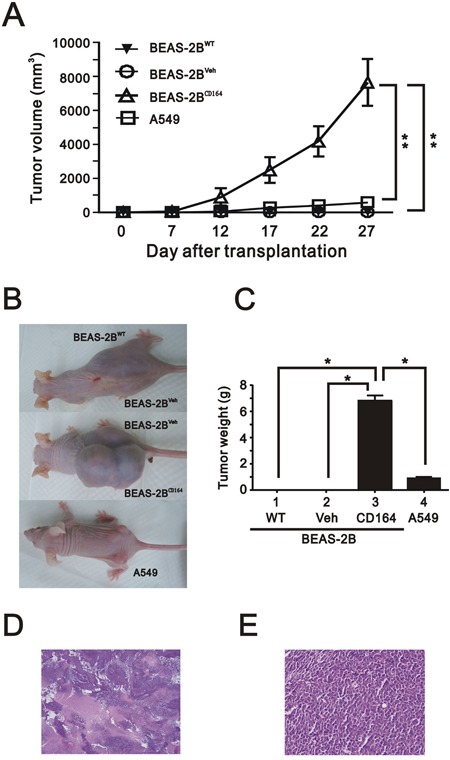
The effect of overexpression of CD164 on tumorigenicity in the xenografted mice **A**. The tumor growth of A549 cells, BEAS2B^CD164^ cells, BEAS2B^Veh^ cells and BEAS2B^WT^ cells in nude mice (n=6, each group) were recorded. The results represented the means ± SEMs. **P < 0.01 indicated statistical significance as compared with A549 cells, BEAS2B^Veh^ cells, and BEAS2B^WT^ cells. **B**. Gross appearance of mice subcutaneously injected with A549 cells, BEAS2B^CD164^ cells, BEAS2B^Veh^ cells and BEAS2B^WT^ cells. **C**. Quantitative analysis of the tumor weight of A549 cells, BEAS2B^CD164^ cells, BEAS2B^Veh^ cells and BEAS2B^WT^ cells. *P < 0.05 indicated statistical significance as compared with A549 cells, BEAS2B^Veh^ cells, and BEAS2B^WT^ cells. Representative H & E stained sections of tumors derived from BEAS2B^CD164^ cells at low (40x) **D**. and higher magnification (400x) **E**.

To identify whether CD164 molecule might be involved in the tumorigenesis of lung cancer *in vivo*, subcutaneous injections of A549 (as a positive control), BEAS2B^WT^, BEAS2B^Veh^, and BEAS2B^CD164^ cells were commenced in nude mice to monitor tumor formation. No visible tumors were in mice injected with either BEAS2B^WT^ or BEAS2B^Veh^ cells at 4 weeks following implantation. Tumor volumes and weight formed by BEAS2B^CD164^ cells exhibited a significantly greater tumor cell growth than A549 and BEAS2B^Veh^ cells (p<0.001) (Figure 3A–3C). Hematoxylin and eosin staining of tumor from mice showed poorly differentiated hyperchromatic tumor cells with increased mitosis and prominent nucleoli (Figure 3D and 3E). Taken together, CD164 expression might be important in the lung epithelial cell tumorigenesis.

### CD164 overexpression induces stem cell-like properties

**Figure 4:**
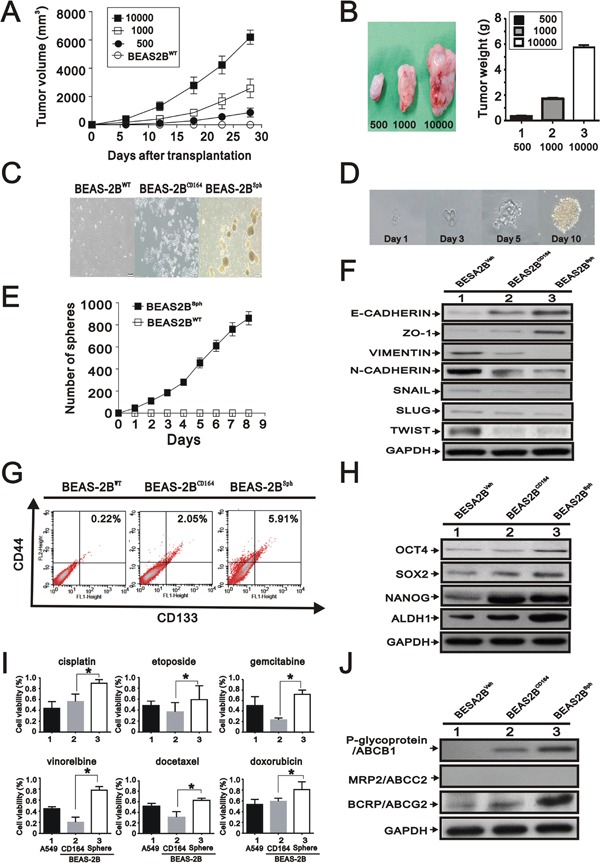
The effect of overexpression of CD164 on the stem cell-like properties **A**. Representative tumor-growth curves of xenografts derived from different cell numbers (n=5, each group). **B**. Quantification of tumor weight formed by different cell numbers of BEAS2B^CD164^ cells. All values were expressed as mean ± SEM. *P < 0.05 indicates statistical significance. **C**. Representative images of BEAS2B^WT^, BEAS2B^CD164^ and BEAS2B^sph^ cells. **D**. One spheroid cell generated from single-cell culture in 96-well plates. **E**. Time course of primary sphere formation after culture in ultralow plate for 8 days. The results were the means ± SEMs of three independent experiments. **F**. Immunoblotting analysis of the indicated proteins in BEAS2B^Veh^, BEAS2B^CD164^, and BEAS2B^sph^ cells. **G**. Flow cytometry for analyzing the expression of CD133 and CD44. **H**. Immunoblotting analysis of stem cell makers in BEAS2B^Veh^, BEAS2B^CD164^, and BEAS2B^sph^ cells. **I**. The chemoresistance of BEAS2B^sph^ and BEAS2B^sph^ cells were performed using MTT assay. All values were expressed as mean ± SEM. *P < 0.05 indicated statistical significance. **J**. Immunoblotting analysis of ABC transporters in BEAS2B^Veh^, BEAS2B^CD164^, and BEAS2B^sph^ cells.

We reasoned that the tumorigenicity along with change of morphological features may be associated with the tumor-initiating potential of BEAS2B^CD164^ cells, because these cells exhibited spheroid body formation in culture. To determine whether CD164 overexpression increased tumor initiation ability, different cell numbers of BEAS2B^CD164^ cells were implanted into nude mice. Our findings showed a substantial difference in tumorigenic properties (Table 2 and Figure 4A). When as few as 500 colonies of BEAS2B^CD164^ cells were subcutaneously injected into nude mice, these cells were able to form tumors (3/5 sites); however, no tumor formation was observed in BEAS2B^Veh^ cells. BEAS2B^CD164^ cells had a significant increase in tumor initiation compared with BEAS2B^Veh^ cells (Figure 4B). Spheroid formation was an important index among the major properties of stem cells that represented the self-renewal capacity. Hence, BEAS2B^CD164^ cells were cultured in serum-free media supplemented with epidermal growth factor (EGF) and basic fibroblast growth factor (bFGF). After 3 weeks of culture, adherent cells began exhibiting spheroid (BEAS2B^sph^) cell characteristics (Figure 4C). We passaged the sphere generated from a single BEAS2B^sph^ cell several times in 96-well plates using the limiting dilution method. We found that spheres originated from single cells rather than cell aggregation, thus suggesting the definitive evidence of the existence of self-renewing cells (Figure 4D). A gradual increase of sphere growth was observed over time for a given number of sphere cells (Figure 4E). We examined the expression of epithelial makers (E-CAHERIN), mesenchymal makers (VIMENTIN and N-CAHERIN), and EMT-associated transcription factors (SNAIL, SLUG, and TWIST) in the BEAS2B^Veh^, BEAS2B^CD164^, and BEAS2B^sph^ cells via Western blot analysis. Increased epithelial marker levels, decreased mesenchymal maker levels, and decreased EMT-associated transcription factors levels were noted in BEAS2B^sph^ cells (Figure 4F), indicating that the MET was driven by CD164 overexpression.

**Table 2:** Comparison of tumorigenic cell doses between BEAS2B^Ve^^h^ and BEAS2B^CD164^ cell

Cell type	Cell number	Number of tumors/number of injections	Tumor onset time (weeks)
BEAS2B^Veh^	10000	0/2 (0%)	-
	500	3/5 (60%)	3-4
BEAS2B^CD164^	1000	3/3 (100%)	2-3
	10000	6/6 (100%)	2

Emerging evidence indicated that CD164 was found to function as a receptor that increased tumor metastasis and hematopoietic stem cell trafficking [23]. Based on the spheroid feature and tumor initiation property, we examined whether several cancer stem cell makers were expressed in BEAS2B^CD164^ cells. Analysis of CD133 and CD44 protein expression by flow cytometry showed that these proteins in the BEAS2B^CD164^ cells and BEAS2B^sph^ cells were higher than BEAS2B^Veh^ cells (Figure 4G). Expression of specific stem cell makers, including Nanog, Oct4, Sox-2, and ALDH1, were elevated in the BEAS2B^CD164^ and BEAS2B^sph^ cells compared with BEAS2B^Veh^ cells (Figure 4H). These findings demonstrated that tumor initiation ability and stem cell-like properties were enhanced by CD164 overexpression.

### BEAS2B^sph^ possesses higher chemotherapeutic drug resistance

There had been increasing evidences that CSCs were involved in drug resistance, tumor regeneration, metastasis, and relapse of cancers [5, 6]. We analyzed the sensitivity of adherent (BEAS2B^CD164^) cells and spheroid (BEAS2B^sph^) cells toward cisplatin, etoposide, gemcitabine, vinorelbine, docetaxel, and doxorubicin, in comparison to A549 cells to test the property of drug resistance. Each chemotherapeutic drug was administered at a concentration inhibiting the A549 cell viability by 50% [12]. In all chemosensitivity tests using cisplatin, etoposide, gemcitabine, vinorelbine, docetaxel, and doxorubicin, BEAS2B^sph^ cells had higher chemotherapeutic drug resistance than BEAS2B^CD164^ cells (Figure 4I). Three ATP-binding cassette (ABC) drug transporters, ABCB1 (p-glycoprotein/MDR1), ABCC1 (MRP1), and ABCG2 (BCRP), have been reported to be critical determinants in the cancer drug resistance [24]. The increased expression of ABCG2 and ABCB1 in BEAS2B^sph^ cells was observed as opposed to BEAS2B^CD164^ cells (Figure 4J). Collectively, these results showed that CD164 expression might be involved in the drug resistance through the induction of ABCG2 and ABCB1.

### CD164 expression increases the CXCR4 and Akt signaling and its downstream factor mTOR

**Figure 5:**
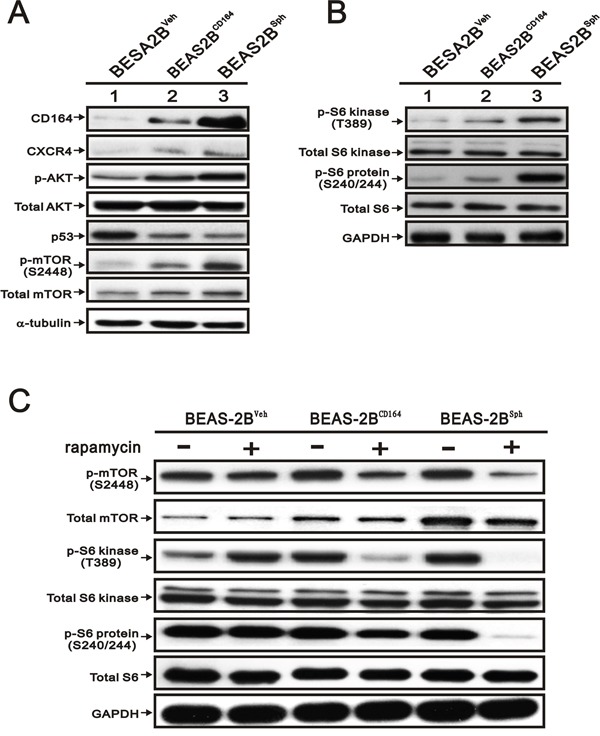
The effect of CD164 overexpression on the PI3K/Akt/mTOR pathway inBEAS-2B cell **A**. Immunoblotting analysis was utilized to determine the protein levels of CD164, CXCR4, AKT, p53, and mTOR in BEAS2B^Veh^, BEAS2B^CD164^ and BEAS2B^sph^ cells. **B**. Immunoblotting analysis of the downstream signaling molecules of mTOR in BEAS2B^Veh^, BEAS2B^CD164^ and BEAS2B^sph^ cells. **C**. The effect of rapamycin on mTOR pathway in BEAS2B^Veh^, BEAS2B^CD164^ and BEAS2B^sph^ cells with vehicle or rapamycin pretreatment.

Recent publications addressed that CD164 molecule activated with CXCR4 and further increased cell proliferation, invasion, migration, and metastasis via activation of the Akt signaling pathways [20, 23, 25]. We further elucidated whether overexpression of CD164 promoted the CXCR4 expression and activated the Akt signaling pathway. Compared with BEAS2B^Veh^ cells, there were significantly higher expressions of CXCR4 and pAkt^Ser-473^ in BEAS2B^CD164^ and BEAS2B^sph^ cells (Figure 5A). Increasing evidence suggested that alterations of phosphatidylinositol-3-kinases (PI3K)/Akt/mTOR pathway were identified and involved in the pathogenesis of lung cancer for therapeutic strategy [26]. In the present study, increased phosphorylation of mTOR at Ser-2448 (S2448) in BEAS2B^CD164^ and BEAS2B^sph^ cells was observed (Figure 5B). mTOR, a serine/threonine protein kinase increased cell growth, proliferation, motility, survival, protein synthesis, autophagy, and transcription, existed in two distinct multi-protein signaling complexes, such as mTORC1 and mTORC2 [27]. We examined the phosphorylation statuses of downstream effectors of mTOR via immunoblotting, because the regulation of translation in the mTORC1 functions was characterized in relation to oncogenesis. Phosphorylation of several downstream signaling molecules, including p70/p85 S6 kinase (at site Thr389, T389), and S6 ribosomal protein (at site Ser240/244, S240/244), was activated in BEAS2B^CD164^ and BEAS2B^sph^ cells (Figure 5B). Moreover, the phosphorylation of p70/p85 S6 kinase and S6 ribosomal protein was decreased when BEAS2B^CD164^ and BEAS2B^sph^ cells were co-incubated with rapamycin (an allosteric mTOR inhibitor, 30 nM) but not in the BEAS2B^Veh^ cells (Figure 5C). These data showed that mTOR was an important downstream factor in CD164 induced CXCR4 and Akt expression.

### Rapamycin diminished the colony formation and sphere formation *in vitro*

**Figure 6:**
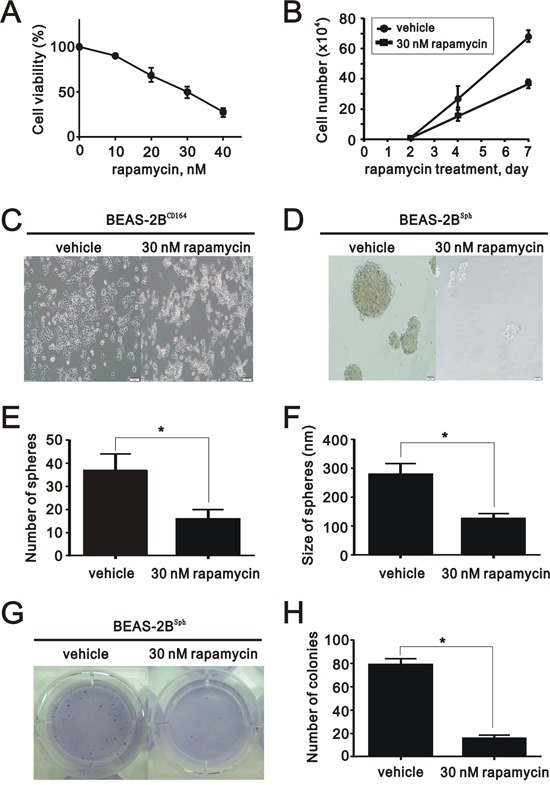
The effect of rapamycin on proliferation, colony formation, and sphere formation *in vitro* **A**. The cell viability of BEAS2B^CD164^ cells treated with different concentrations of rapamycin was measured. The results were the means ± SEMs of three independent experiments. **B**. The cell numbers of BEAS2B^CD164^ cells treated with and without 30 nM rapamycin were recorded at the indicated time points. **C**. Representative photos of BEAS2B^CD164^ cells untreated and treated with 30 nM rapamycin for three days. **D**. Cellular morphology of BEAS2B^sph^ cells untreated and treated with 30 nM rapamycin for three days. **E**. The number of spheres formed in the ultralow plate was recorded in triplicate plates. All values were expressed as mean ± SEM. *P < 0.05 indicates statistical significance. **F**. The size of spheres formed in the ultralow plate was determined in triplicate plates. All values were expressed as mean ± SEM. *P < 0.05 indicates statistical significance. **G**. Representative images of the anchorage independent growth of BEAS2B^sph^ cells untreated and treated with 30 nM rapamycin. **H**. Quantification of anchorage independent growth of BEAS2B^sph^ cells untreated and treated with 30 nM rapamycin. All values were expressed as mean ± SEM. *P < 0.05 indicates statistical significance.

We treated BEAS2B^CD164^ cells with rapamycin and analyzed cell viability via MTT assay. The results indicated rapamycin significantly diminished BEAS2B^CD164^ cell viability (Figure 6A). The half inhibitory concentration of rapamycin (50% viability) was 30 nM. Using 30 nM rapamycin, the growth rate of BEAS2B^CD164^ cells was reduced by approximately 50% after 7-day incubation (Figure 6B). The implementation of rapamycin in the BEAS2B^CD164^ cells did not induce change of cell morphology (Figure 6C). BEAS2B^CD164^ cells formed spheres that reached 200-300 μm in diameter after 10-14 days. The application of 30 nM rapamycin impeded sphere formation (BEAS2B^sph^ cells) in the number and size (Figure 6D–6F). Meanwhile, significant reductions in colony formation efficiency were observed in anchorage-independent growth of BEAS2B^sph^ cells treated with 30 nM rapamycin (Figure 6G and 6H). This finding further supported mTOR, an effector protein of CD164 induced Akt signaling, might be involved in the self-renewal of cancer stem-like cells.

### Rapamycin impeded the growth of CD164-overexpressed BEAS2B cells in xenotransplant mice

**Figure 7:**
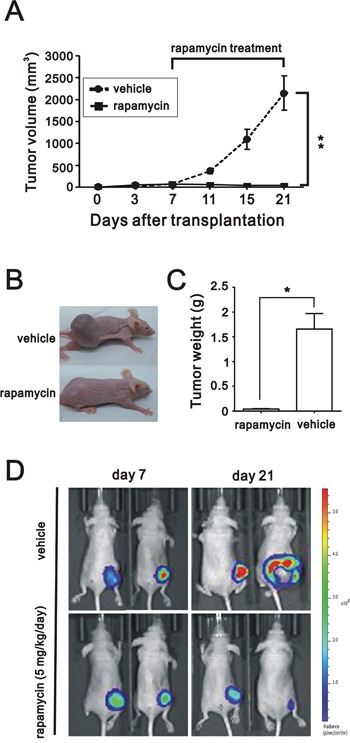
The effect of rapamycin on *in vivo* growth of xenograft BEAS2B^CD164^ cells **A**. Tumor growth of BEAS2B^CD164^ cells xenograft was untreated and treated with 5 mg/kg/day rapamycin. **B**. Photomicrographs of the xenografted mice treated and untreated with rapamycin (5 mg/kg/day). **C**. Quantitative analysis of the tumor weight in control group and rapamycin group. *P < 0.05 indicated statistical significance as compared with rapamycin group. **D**. Bioluminescent images of control group and rapamycin group using an IVIS spectrum after 7 days and 21 days of cells implantation.

To focus on the inhibitory effect of rapamycin in xenotransplant mice, intraperitoneal administration with rapamycin at 5 mg/kg/day in nude mice bearing subcutaneous xenograft tumors derived from BEAS2B^CD164^ cells was commenced. After 3 week of observation, final volume of BEAS2B^CD164^ cells derived tumors was significantly reduced after daily intraperitoneal injections of rapamycin compared to control group (P<0.001) (Figure 7A). The tumor weight in the rapamycin-treated group was much greater than that in the control group (Figure 7B and 7C). As monitored by *in vivo* bioluminescent imaging, the application of rapamycin largely suppressed tumor volume in tumor-bearing mice as opposed to the control group (Figure 7D).

### Increased the expression of CD164 in the lung tumor spheroid cells

**Figure 8:**
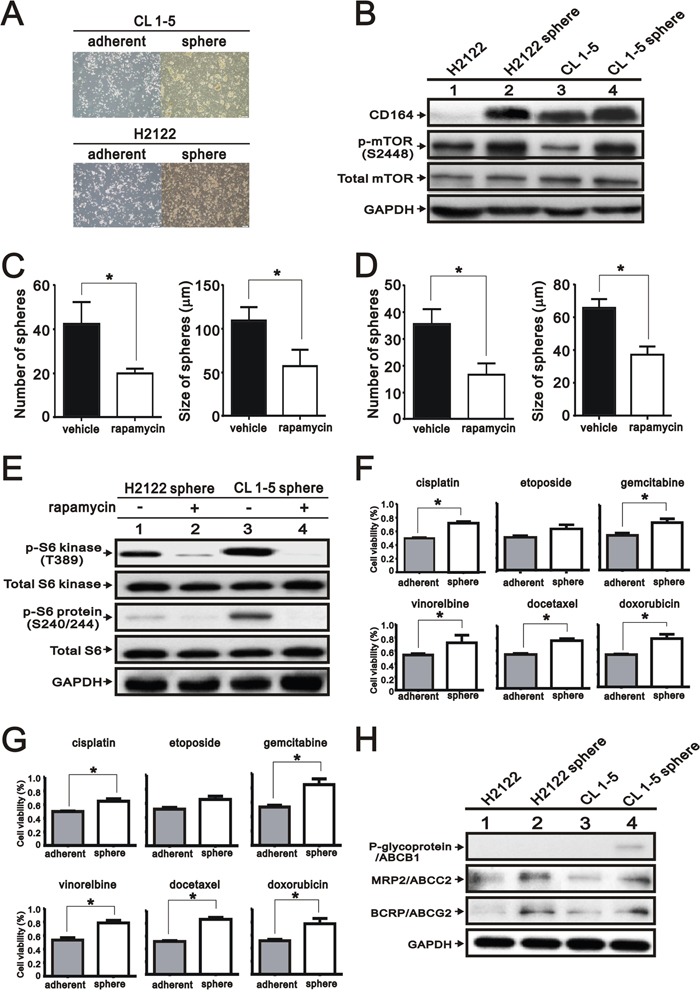
Expression of CD164 in the lung tumor sphere cells **A**. Representative photographs of spheroid cells and adherent cells of H2122 and CL 1-5. **B**. Immunoblotting analysis was utilized to determine the protein levels of CD164 and mTOR in the spheroid cells of H2122 and CL 1-5. **C**. The number and size of CL 1-5 spheres formed in the ultralow plate were determined in the absence or presence of 30 nM rapamycin. All values were expressed as mean ± SEM of three independent experiments. *P < 0.05 indicated statistical significance. **D**. The number and size of H2122 spheres formed in the ultralow plate were determined in the absence or presence of 30 nM rapamycin. All values were expressed as mean ± SEM of three independent experiments. *P < 0.05 indicated statistical significance. **E**. Immunoblotting analysis was used to determine the mTOR pathway in the spheroid cells of H2122 and CL 1-5 treated with vehicle or rapamycin. **F**. The chemo-resistances of H2122 spheroid cells and adherent cells were performed using MTT assay. All values are expressed as mean ± SEM of three independent experiments. *P < 0.05 indicated statistical significance. **G**. The chemo-resistances of CL 1-5 spheroid cells and adherent cells were performed using MTT assay. All values were expressed as mean ± SEM of three independent experiments. *P < 0.05 indicates statistical significance. **H**. Immunoblotting analysis of ABC transporters in the spheroid cells and adherent cells of H2122 and CL 1-5.

To identify the involvement of the CD164 on the spheroid cell formation from lung cancer cell lines, we cultured H2122 and CL 1-5 cells under stem cell growth medium in 96-well plates via the limiting dilution method. The formation of spheroid cells was found derived H2122 and CL 1-5 adherent cells after 2 weeks (Figure 8A). Notably, the abundances of CD164 and phosphorylation of mTOR were enhanced in spheroid cells derived from H2122 and CL 1-5 cells as compared to the adherent cells of H2122 and CL 1-5 cells (Figure 8B). Regarding spheroid cells derived from H2122 and CL 1-5 cells, treatment with 30 nM rapamycin had the ability to inhibit sphere formation in the number and size (Figure 8C and 8D). Immunoblotting analysis showed that p70/p85 S6 kinase (T389) and S6 ribosomal protein (S240/244) levels were reduced in rapamycin-treated groups compared with control groups (Figure 8E). Moreover, spheroid cells derived from H2122 and CL 1-5 had higher resistance to chemotherapeutic drugs than adherent H2122 and CL 1-5 cells, respectively (Figure 8F and 8G). Compared with adherent H2122 and CL 1-5 cells, spheroid cells derived from H2122 and CL 1-5 cells expressed higher levels ofABCG2 and ABCC2 (Figure 8H).

### Depletion of CD164 expression by small hairpin RNA (shRNA) decreased lung cancer cell proliferation

**Figure 9:**
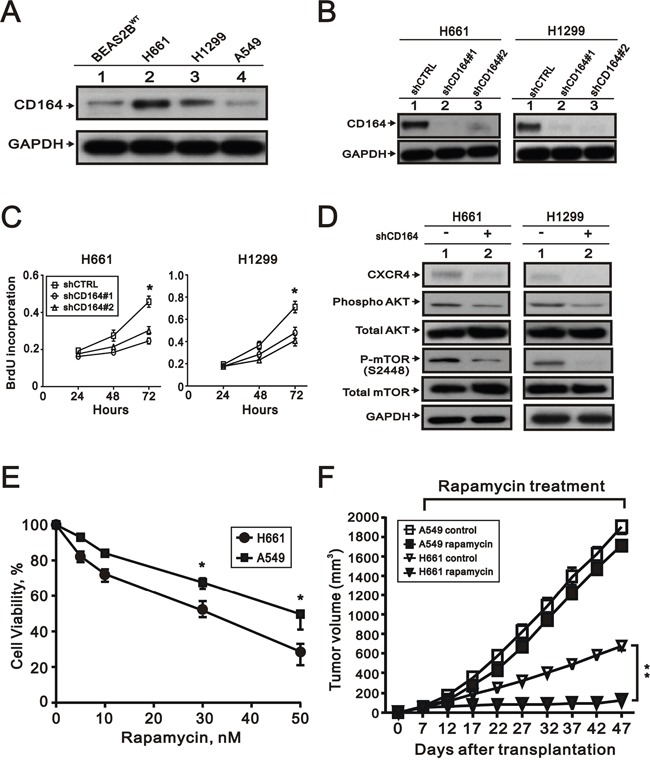
The effect of CD164 inhibition on lung cancer cells **A**. CD164 expression was measured by western blot analysis in the four indicated cell lines. **B**. Western blotting showed the efficacy of the small hairpin RNA (shRNA)-lentiviral approach to decrease CD164 expression in the H661 and H1299. **C**. Proliferation of H661 and H1299 cells infected with control (shCTRL), CD164 shRNA#1 (shCD164#1), and CD164 shRNA#2 (shCD164#2) were evaluated by the BrdU proliferative assay. The results were the means ± SEMs of three independent experiments. *P < 0.05 indicated statistical significance. **D**. Immunoblotting analysis was used to determine the protein levels of CXCR4, AKT, and mTOR in H661(shCD164) and H1299(shCD164) cells. **E**. The cell viability of H661 and A549 cells treated with different concentrations of rapamycin was recorded. *P < 0.05 indicated statistical significance. The results were the means ± SEMs of three independent experiments. **F**. Tumor growths of H661 and A549 cells xenograft were treated with vehicle or rapamycin.

Compared with BEAS2B^WT^, we performed the Western blot analysis to examine the expression of CD164 in three lung cancer cell lines, including H661, H1299, and A549 (Figure 9A). The expressed CD164 levels of H661 and H1299 cells were higher than BEAS2B^WT^ cells. To determine the effects of CD164 on lung cancer cell growth, two CD164 knockdown stable clones were successfully established and identified by the Western blot analysis (Figure 9B). The cell proliferation rates of H661 and H1299 were markedly decreased by CD164 shRNA (shCD164) via the BrdU proliferation assay (Figure 9C). In H661 and H1299 lung cancer cells, Western blot analysis demonstrated that diminished CD164 expression resulted in decreased Akt and mTOR phosphorylation, and decreased CXCR4 activation (Figure 9D).

### Direct comparisons of higher CD164 expression and lower CD164 expression of lung cancer cell lines *in vitro* and *in vivo*

Among the three lung cancer cell lines, the expressed CD164 levels of H661 and H1299 cells were higher than BEAS2B^WT^, while the expressed CD164 level of A549 cells were lower than BEAS2B^WT^ (Figure 9A). H661 cells had the highest level of CD164 expression and A549 cells had the lowest level of CD164 expression in our current work. In order to direct comparisons of higher CD164 expression of H661 cells and lower CD164 expression of A549 cells, we tested the anti-proliferative effects of rapamycin on each cell type by MTT assay (Figure 9E). A549 cells were more resistant to rapamycin than H661 cells. In the xenotransplant mice model, rapamycin significantly inhibited the xenografts growth of H661 cells but not the xenografts growth of A549 cells (Figure 9F).

## DISCUSSION

**Figure 10:**
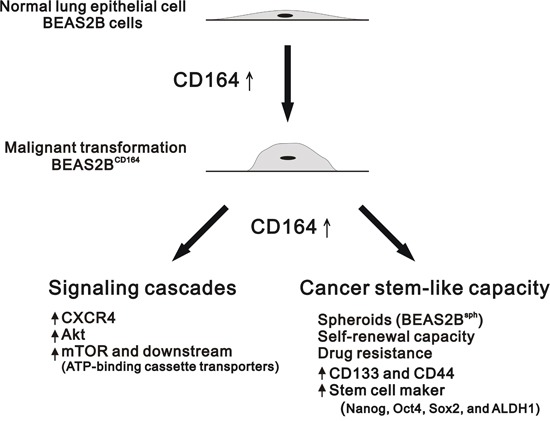
Schematic overview of the CD164 induction for the tumorigenesis of normal lung epithelial cells The overexpression of CD164 in BEAS2B cells leads to tumorigenicity in the xenografted mice and induces the stem cell-like property and drug resistance. CD164 promoted lung tumor-initiating cells with stem cell properties and induced tumor growth and drug resistance through Akt/mTOR signaling.

To the best of our knowledge, this is the first study to evaluate association between CD164 expression and the clinical characteristics of patients with lung cancer. CD164 has emerged as a multitasking protein that functions as a hematopoietic stem cell surface marker, a CXCR4 promoter activity-enhancing transcription factor, and a stem cell-specific maker inducer [16, 17, 22]. In the present study, we demonstrated that CD164 overexpression significantly increased tumorigenicity and stem cell-like properties in BEAS2B cells. Compared with BEAS2B^Veh^ cells, there were significantly higher expressions of CXCR4, phosphorylated Akt, and phosphorylated mTOR in BEAS2B^CD164^ and BEAS2B^sph^ cells. In addition, rapamycin hinders the cell proliferation and sphere formation of BEAS2B^CD164^ cells *in vitro* and impedes the tumor growth *in vivo*. Hence, we propose that CD164 overexpression increases cell proliferation and chemoresistance via the PI3K/Akt/mTOR pathway, resulting in the conversion of normal human bronchial epithelial cell to tumor-initiating cells (Figure 10).

Disclosing the cell origin of different subtypes in lung cancers and elucidating the molecular biology of transformation is an imperative prerequisite to help the development of new therapeutic treatment of lung cancer. To address this issue, a steadily growing amount of work was beginning to handle lung cancer through oncogene-related transformation of normal lung cells, including H-ras [28], K-ras [29], Trp53 and Rb1 [30], PIK3CA, cyclin D1, or a dominant-negative form of LKB1 [31]. Similarly, our previous study in which CD164 overexpression contributes to p53 suppression, suggesting that CD164 molecule may counteract p53 to facilitate and control tumorigenesis. In addition, we analyzed the clinical relevance of CD164 from a public online database PRECOG (PREdiction of Clinical Outcomes from Genomic profiles, available at http://precog.stanford.edu). At least, two studies showed that higher CD164 mRNA expression in patients with lung adenocarcinoma and squamous cell lung cancer might significantly correlate with worse overall survival (HR =2.13; HR =1.68) in the five Kaplan-Meier survival curves from PRECOG database [32, 33]. Hence, CD164 might be the potential molecule to develop as a new therapeutic target for lung cancer.

Several research works proposed that increased CXCR4 expression was associated with enhanced metastatic potential of lung cancer cells and contributed to increase in their self-renewal activity, which may be key regulator of tumor invasiveness leading to local progression and tumor metastasis [34]. CXCR4^+^ cells from the Lewis lung carcinoma cell line exhibit cancer metastatic stem cell characteristics [11]. CD133^-^CXCR4^+^ovarian cancer cells expressed less sensitive to cisplatin and higher level of ABCG2 transporters than CD133^-^CXCR4^-^ ovarian cancer cells displaying a drug resistance phenotype [35]. In adult acute leukemia, ABCG2 expression can be elevated by the inactivation of phosphatase and tensin homolog (PTEN) protein, achieved in this study by reversing the PI3K/Akt pathway inhibition [36]. Consistent with our findings, BEAS2B^sph^ cells had elevated expression of CXCR4 receptor and stem cell makers accompanied by drug resistance and formation of sphere. Collectively, it was tempting to speculate that CD164 activated with CXCR4 to exhibit stem cell-like properties and drug resistance via the PTEN/PI3K/Akt pathway and ABCG2 expression.

Over the last five years, several studies have focused on the roles of mTOR signaling activation in CSCs in relationship with the tumorigenesis and drug resistance. Research involving leukemic stem cells showed that rapamycin prevented leukemia development, implying an important role for mTORC1 in leukemogenesis [37]. Intriguingly, when rapamycin was administered in a model of pancreatic carcinoma, mTOR was involved in the maintenance of stem-like property of pancreatic cancer stem cells and mTOR inhibitor significantly impaired *in vivo* growth of xenografted pancreatic carcinoma [38]. Similar results have been reported that rapamycin decreased glioma stem cell activity and temozolomide resistance through SOX2/SOX9 expression [39]. In addition, the pharmacological PI3-kinase inhibitor LY294002 and the downstream mTOR kinase inhibitor rapamycin directly inhibited ABCG2 function, thereby possibly decreasing the degree of chemoresistance [40]. Consistent with our findings, rapamycin diminished the stem cell-like capacity of BEAS2B^CD164^ cells *in vitro* and overtly inhibited subcutaneous xenograft tumors. PI3K/Akt/mTOR signaling activation was required for maintenance of stem-like homeostasis, tumorigenesis, and chemoresistance in the CD164 overexpression of bronchial epithelial cell. In case of lung cancer patients with chemoresistance and high expression of CD164, therapeutic targeting of mTOR might be considered as an alternative potential therapy.

The major ABC superfamily transporters associated with multidrug resistance development were ABCB1, MRP2/ABCC2, and BCRP/ABCG2, which were often enriched with cells cancer stem cell-like phenotypes [41, 42]. In terms of NSCLC, overexpression of ABCG2 had been reported to confer drug resistance to various chemo-therapeutic drugs [43]. Intriguingly, ABCG2 might serve as a novel biomarker of CSCs because of the ABCG2 side population phenotype and the conserved expression of ABCG2 in stem cells [44]. In our study, the increased expression of ABCG2 was consistently found in the BEAS2B^sph^ cells, H2122 sphere, and CL 1-5 sphere. However, we were unable to confirm which one of ABC transporters was responsible for the drug resistant phenotype, because individual knockdown of ABC transporters was not performed. We proposed that the ABC family of membrane transporters possibly accounted for the drug resistance when spheroid cells or CSCs were formed. Our study raises the interesting possibility warrant thorough investigation and prospective testing.

EMT endowed cells with migratory and invasive properties, induces stem cell properties, prevents apoptosis and senescence, and contributes to immunosuppression [45]. MET promoted epithelial gene expression and facilitates colonization during tumor initiation at metastatic sites [46]. The phenomenon of EMT and MET was a dynamic change in cell behavior during cancer cell dissemination and metastatic colonization. In our study, BEAS2B^CD164^ and BEAS2B^sph^ cells had increased epithelial marker level, decreased mesenchymal maker expression, cell-cell adhesion, and increased proliferation, implying a MET. In agreement with previous studies, characteristic features of MET included elevated E-cadherin, cell-cell adhesion, and increased proliferation [47]. In addition, emerging evidences addressed that epithelial markers [48–50] but not mesenchymal markers [51, 52] predicted metastasis and poor survival outcome in patients with breast cancer. Notably, migration or formation of mammospheres and increased tumorigenicity are often not associated with expression of mesenchymal genes but rather with enrichment for epithelial gene expression in several breast cancer cell lines [53, 54]. Because complicated regulatory networks controlled stemness and cellular plasticity in cancer stem cells, whether CD164 expression might directly promote MET remained to be investigated.

In conclusion, we found that CD164 overexpression in normal lung epithelial cells may promote tumorigenicity, stem cell-like properties, and chemoresistance via the CXCR4/AKT/mTOR pathway. Rapamycin inhibited sphere formation *in vitro* and xenograft tumor growth from higher CD164 expression cells *in vivo*. We disclosed that CD164 might be an imperative molecule linking tumor-initiating and chemoresistance because it might activate either the downstream signaling of mTOR or ABC transporters. The model described here provided the CD164 expression in lung tumorigenic cancer-initiating cells may represent a key therapeutic strategy for future lung cancer treatment.

## MATERIALS AND METHODS

### Ethics statements

All experiments were performed based on the Institutional Ethical Guidelines. All animal studies were approved (IACUC No. 13-294) by the Laboratory Animal Center (accredited by the Association for Assessment and Accreditation of Laboratory Animal Care International (AAALAC)) of National Defense Medical Center in accordance with the guidelines of the Institutional Animal Care and Use Committee.

### Cell culture and reagents

Normal human bronchial epithelial BEAS-2B cells, human lung cancer H2122 cells, and A549 cells were derived from the American Type Culture Collection (ATCC, Manassas, VA, USA). The lung adenocarcinoma cell lines (CL1-5) were a gift from Professor Pan-Chyr Yang (National Taiwan University) and established in the National Health Research Institutes laboratory [55]. All cells were cultured in Roswell Park Memorial Institute (RPMI) 1640 medium supplemented with 1% penicillin/streptomycin and 10% fetal bovine serum in a humidified atmosphere containing 5% CO_2_ at 37°C. The culture medium was replaced twice weekly.

CD164 polyclonal antibody was purchased from R&D systems (Oxford, UK). CXCR4 antibody was obtained from Abcam (Cambridge, United Kingdom). Anti-phospho-mTOR (Ser2448), anti-mTOR, anti-phospho-p70/p85 S6 Kinase (S6K, T389), anti-p70/p85S6 Kinase (S6K), anti-phospho-S6 ribosomal protein (S240/244), anti-S6 ribosomal protein, anti-phospho-Akt (T308), anti-phospho-Akt (S473), anti-Akt, anti-E-CAHERIN, anti-ZO-1, anti-VIMENTIN, anti-N-CAHERIN, anti-SNAIL, anti-SLUG, anti-TWSIT, anti-Oct 4, anti-SOX 2 and anti-ALDH1 antibodies were purchased from Cell Signaling (Cell Signaling Technology, Beverly, MA, USA). The chemical agents, including rapamycin, cisplatin, etoposide, gemcitabine, vinorelbine, docetaxel, and doxorubicin, were obtained from Sigma Aldrich (Sigma Aldrich Corp., St. Louis, MO, USA).

### Tissue microarray

Two sets of commercial tissue microarrays were used in the study (LC1923 and LC2085b, US Biomax Inc., MD, USA). Surgical resection of tissue samples consisted of 20 normal lung tissues, 138 adenocarcinomas, 150 squamous cell carcinomas, 12 large cell carcinomas, 37 small cell lung cancers, and 23 other types (bronchioalveolar carcinoma (n=7), adenosquamous carcinoma (n=5), atypical carcinoid (n=4), mucinous carcinoma (n=4), papillary adenocarcinoma (n=2), and sarcomatoid carcinoma (n=1)).

Each tissue was evaluated for staining intensity and percentage of cells staining. An H-score was generated as the sum of the products of each staining intensity multiplied by the percentage of cells staining [56].

### Flow cytometric analysis

After washing by cooled 1×PBS, the cells were resuspended in buffer with PBS, 0.5% BSA, and 2 mmol/L EDTA. Based on antibody concentrations recommended by manufacturer's instructions, the cells (2×10^5^) were incubated with the following antibodies, including anti-human CD164-APC (R&D Systems, Minneapolis, MN, USA), CD133-PE (Miltenyi Biotec, Auburn, California, USA), and CD44-FITC (eBioscience, San Diego, CA, USA). Appropriate isotype antibodies were chosen as controls. Data were analyzed by the FACSCalibur flow cytometer (Becton Dickinson, Franklin Lakes, New Jersey, USA). Cell debris was excluded from the analysis according to scatter signals.

### Sphere formation assay

Spheroid cells were cultured in serum-free Dulbecco's modified Eagle's medium supplemented with 20 ng/ml epidermal growth factor (Sigma Aldrich Corp., St. Louis, MO, USA) and 20 ng/mL basic fibroblast growth factor (Sigma Aldrich Corp., St. Louis, MO, USA). The Spheroid cells were dissociated and diluted to a density of 500-1000 cells/ml by limiting dilution assay. A 2 μl aliquot was seeding to each well of 96-wellultra-low attachment plates in 100 μL of serum free medium and cultured for up to 7 days. Fresh medium was replenished every 3 days. The numbers and sizes of spheres were recorded during this period.

### Soft agar colony formation assay

Soft agar colony formation assay was performed to investigate the ability to exhibit anchorage-independent cell growth. A total of 1×10^4^ cells were harvested and suspended in 2 ml of medium containing 0.35 % agar, and these cells were seeded into6-well culture plates with 2ml of 0.5 % agar base layer. Colony formation was analyzed via crystal violet staining after 3-week of culture at 37°C with 5% CO_2_. Colony numbers were counted by microscopic examination.

### MTT cell viability assay

Cell viability was evaluated by 3-(4,5-Dimethyl-2-thiazolyl)-2,5-diphenyl-2H- tetrazolium Bromide (MTT) assay. Membrane-permeable yellow dye was reduced by mitochondrial reductases in living cells to form the dark blue product, MTT-formazan. Cells were seeded at a density of 1 ×10^4^ cells/well in a 96-well plate and cultured overnight. After 4 hours incubation at 37°C with 10 μl of 5 mg/ml of MTT (Millipore, Billerica, MA, USA), DMSO was added for crystals solubilization. Spectrophotometric measurement of MTT-formazan at 540 or 570 nm allowed cell viability quantitation.

### Immunoblotting

After the washing with ice-cold PBS twice, the cells were prepared with RIPA lysis buffer (150 mM NaCl, 1% NP40, 0.5% DOC, 50 mM Tris-HCl at pH 8, 0.1% SDS, 10% glycerol, 5 mM EDTA, 20 mM NaF, and 1 mM Na_3_VO_4_) containing protease inhibitors. Cell lysates were centrifuged at 15,000 g for 20 minutes at 4°C, and the clear supernatant was removed and placed into new Eppendorf tubes. After the protein concentration were quantitatively determined by BCA assay (Pierce Biotechnology Inc, Thermo-Scientific, Rockford, IL, USA), the proteins were separated by sodium dodecyl sulfate-polyacrylamide gel electrophoresis and transferred onto a polyvinylidene difluoride membrane (Millipore Corp., Bedford, MA, USA). The membranes were blocked with 5% milk in Tris-buffered saline containing 0.05% Tween-20 for 1 hour at room temperature. After the blots were incubated overnight at 4°C, specific primary antibodies and appropriate secondary antibodies were used. The proteins were visualized on X-ray film using the standard enhanced chemiluminescence procedure.

### Immunohistochemical staining

For immunohistochemical procedures, sections were deparaffinized with xylene and immersed in graded ethanol and distilled water. Endogenous peroxidase activity was blocked at room temperature by 5-10 minutes of incubation in the 3% H_2_O_2_ in PBS (pH 7.4). After the rehydrated sections were treated with 0.01 M sodium-citrate buffer (pH 6.3) for antigen retrieval at 95°C for 30 minutes, they were incubated with a dilution of 1:100 purified rabbit anti-human CD164 antibody (Sigma Aldrich Corp., St. Louis, MO, USA) for 60 minutes at room temperature. After secondary antibody application, the slides were developed with diamino benzamidine (DAB) and counterstained with hematoxylin. Negative control included omitting the primary antibody, incubating with PBS, and replacing the primary antibody with normal serum. Each tissue was evaluated regarding staining intensity and percentage of cells staining. H-scores were generated as the sum of the products of each intensity category multiplied by the extents of immunoexpression [56].

### Mouse xenograft experiment

Groups of NOD mice aged 5-6 weeks were purchased from BioLASCO Taiwan (Yi-Lan Breeding Center, Yi-Lan County, Taiwan), which was accredited by the Association for Assessment and Accreditation of Laboratory Animal Care International. Experimental mice were housed in a temperature- and humidity-controlled animal room under a 12-hour light/12-hour dark cycle and pathogen-free conditions, with food and water provided ad libitum.

The cells were calculated the cell viability and cell number via trypan blue, and 2×10^6^ cells in HBSS mixed (1:1 volume) with Matrigel (Corning Inc., New York, NY, USA) were subcutaneously injected to the dorsal part of each mouse. Mice were monitored daily for tumor formation. Tumor volumes were measured in two dimensions with a caliper and their volumes were estimated using the indicated standard formula (width^2^ x length x 0.5). At the end of each experiment, the mice were dissected and the tumors were fixed in fresh formaldehyde buffer.

### Bioluminescence imaging and rapamycin treatment

CD164-overexpression BEAS2B cells (BEAS2B^CD164^) transfected by a firefly luciferase cDNA expression vector were kindly provided as a gift by Dr. PW Hsiao (Agricultural Biotechnology Research Center, Academia Sinica, Taipei, Taiwan). Luciferase activity was determined by luciferase activities assay. BEAS2B^CD164^ cells with the highest luciferase activity were selected and used for the *in vivo* animal experiments. Six-week-old nude mice were subcutaneously inoculated into the dorsal flank with 2×10^6^ luciferase-expressed BEAS2B^CD164^ cells. In order to assess the *in vivo* effects of rapamycin, mice were randomly categorized into the following 2 groups (5 mice per group) 7 days after cell implantation: a group that received 5 mg/kg of rapamycin and another group that received vehicle (dimethyl sulfoxide, DMSO) by daily intraperitoneal injection. Mice were anesthetized with isoflurane (Panion & BF Biotech Inc., Taipei, Taiwan). The substrate n-luciferin (Sigma Aldrich Corp., St. Louis, MO, USA) was intra-peritoneally injected at a dose of 150 mg/kg body weight (30 mg/ml) 5 minutes before imaging. Mice were pictured using a non-invasive vivo imaging system (IVIS) (PerkinElmer, UA) coupled to a charge-coupled device camera. Bioluminescence imaging of the implanted tumors and mouse body weights were recorded once per week.

### Lung cancer xenografts and rapamycin treatment

A549 and H661 cells at 5×10^6^ in serum-free medium were subcutaneously into the dorsal flank of six-week-old nude mice. When the xenograft tumors of A549 and H661 cells reached about 100 mm^3^, the mice were randomized into two groups (n=5/group): control group and rapamycin group (daily intraperitoneal injection of 1 mg/kg of rapamycin). The two groups were treated for 40 days, and tumor growth of mice was recorded at five-day intervals.

### Lentiviral infection for CD164 overexpression or knockdown

The human CD164 gene coding sequence was amplified by polymerase chain reaction and subcloned into a pWPXL lentivirus expression plasmid (Addgene, Cambridge, USA) vector along with EcoRI restriction sites. The lentivirus expression plasmid and two packaging plasmids psPAX2 (Addgene) and pMD2.G (Addgene) were co-transfected into human embryonic kidney cells (HEK293T) in serum-free medium using Lipofectamine TM 2000 (Invitrogen Co., Carlsbad, CA), according to the manufacturer's instructions. The lentiviral CD164 shRNA vectors (Clone ID:#1: TRCN0000289234 and #2: TRCN0000310267) and a pLKO control lentiviral vector were obtained from the National RNAi Core Facility in Academic Sinica, Taipei, Taiwan. Cells infected with the lentivirus containing CD164 shRNAs were selected in 2 μg/ml puromycin. Pooled populations of knockdown cells were used for further experiments.

### Drug sensitivity testing

For the spheroid cells, spheres were dissociated by trypsinization with 0.25 % Trypsin and 0.02% EDTA for 2 min at 37 °C and physical separation by pipetting. All adherent and sphere derived cells were incubated in 96-well plates with growth medium at 100 μl per well for 12 hours. All chemotherapeutic drugs were used at the IC_50_ concentration, including cisplatin, etoposide, gemcitabine, vinorelbine, docetaxel, and doxorubicin (Sigma Aldrich Corp., St. Louis, MO, USA). After 24 hours of co-incubation, cell viability was monitored using a colorimetric3-(4,5-Dimethyl- 2-thiazolyl)-2,5-diphenyl-2H-tetrazolium based on the manufacturer's protocol. Regarding drug resistance, the percentage of viability was calculated using the following formula: viable cells (%) = absorbance of drug-treated sample/absorbance of untreated sample×100.

### Statistical analysis

All experimental studies were independently repeated three or more times. Quantitative parameters were expressed as the mean and standard deviation, while qualitative data were presented as number and percentage. Statistical analyses were performed using one-way analysis of variance (ANOVA) and Student's t-test. Two-sided p values less than 0.05 were considered significant.

## Abbreviations

NSCLC: non-small cell lung cancer
CSCs: cancer stem cells
NOD: non-obese diabetic
SCID: severe combined immunodeficiency
CXCR4: C-X-C chemokine receptor type 4
ALDH1: aldehyde dehydrogenase 1
BEAS2B^CD164^: CD164-overexpression BEAS2B cells
BEAS2B^WT^: BEAS-2B wild type
BEAS2B^Veh^: BEAS2B-vehicle cell
BEAS2B^sph^: spheroid cells derived from CD164-overexpression BEAS2B cells
EGF: epidermal growth factor
bFGF: basic fibroblast growth factor
ABC: ATP-binding cassette
ABCB1: p-glycoprotein/MDR1
MRP2/ABCC2: multidrug resistance-associated protein 2
BCRP/ABCG2: breast cancer resistance protein
